# An Improved Model for Nucleation-Limited Ice Formation in Living Cells during Freezing

**DOI:** 10.1371/journal.pone.0098132

**Published:** 2014-05-22

**Authors:** Jingru Yi, Xin M. Liang, Gang Zhao, Xiaoming He

**Affiliations:** 1 Centre for Biomedical Engineering, Department of Electronic Science and Technology, University of Science and Technology of China, Hefei, Anhui, China; 2 Department of Mechanical Engineering, University of Washington, Seattle, Washington, United States of America; 3 Department of Biomedical Engineering, The Ohio State University, Columbus, Ohio, United States of America; University of Zurich, Switzerland

## Abstract

Ice formation in living cells is a lethal event during freezing and its characterization is important to the development of optimal protocols for not only cryopreservation but also cryotherapy applications. Although the model for probability of ice formation (PIF) in cells developed by Toner et al. has been widely used to predict nucleation-limited intracellular ice formation (IIF), our data of freezing Hela cells suggest that this model could give misleading prediction of PIF when the maximum PIF in cells during freezing is less than 1 (PIF ranges from 0 to 1). We introduce a new model to overcome this problem by incorporating a critical cell volume to modify the Toner's original model. We further reveal that this critical cell volume is dependent on the mechanisms of ice nucleation in cells during freezing, *i.e.*, surface-catalyzed nucleation (SCN) and volume-catalyzed nucleation (VCN). Taken together, the improved PIF model may be valuable for better understanding of the mechanisms of ice nucleation in cells during freezing and more accurate prediction of PIF for cryopreservation and cryotherapy applications.

## Introduction

Cryopreservation and cryotherapy are the two typical biomedical applications of cryogenics. On one hand, cryopreservation, the long-term banking of cells and tissues at cryogenic temperatures, is an enabling technology for the eventual success of cell-based medicine including tissue engineering, regenerative medicine, and assisted reproduction [1–8]. On the other hand, cryotherapy (also called cryosurgery or cryoablation) that utilizes freezing to destroy undesired tissues, is attracting more and more attention as a minimally invasive alternative to radical surgical intervention for treating cancer and many other diseases [8–11]. However, if the freezing protocol is not well designed, cryotherapy could end up with incomplete tumor destruction and cancer recurrence ensues [11,12]. Therefore, a good understanding of the response of tumor cells to freezing is of importance and significance for optimizing the freezing protocols to improve the treatment outcome of cryotherapy.

When freezing cell suspension, ice forms in the extracellular water first. Consequently, the concentration of extracellular solution increases, which breaks the balance in chemical potential between water inside and outside of cells. As a result, water either osmoses out of the cells through their plasma membrane (and cells dehydrate) or undergoes phase change to form ice in the cells. Freezing injury emerged in this process is generally believed to be related to these two fates of intracellular water: dehydration or solution effect [13] and intracellular ice formation (IIF) [14,15]. Among the two, IIF is considered to be the most deleterious and it damages the cells in both mechanical and non-mechanical fashions [8]. Therefore, it is important to understand IIF during freezing to either minimize it (for cryopreservation) or maximize it (for cryotherapy).

As with all phase change phenomena, ice formation in cells is a two-step process: nucleation of ice embryos and their subsequent growth. Moreover, in the absence of cryoprotective agents (CPAs), once ice embryos are nucleated in a cell, ice will instantly propagate throughout the whole cell [15,16]. This is because the viscosity is low (or diffusion coefficient is high) in physiologic solutions such as that in living cells, which allows instantaneous growth of ice embryos throughout the tiny cellular space. As a result, IIF in the absence of CPAs (e.g., for cryotherapy applications) is nucleation-limited. Therefore, ice nucleation in cells during freezing has been extensively studied in the past decades both experimentally and by modeling [15–24]. For the latter, the Toner's PIF (Probability of IIF) model [15] has been the most widely used for quantifying IIF that is nucleation-limited [14,25]. This model is based on the catalytic mechanisms including both surface-catalyzed nucleation (SCN) and volume-catalyzed nucleation (VCN) [15,22]. However, this model assumes that apparent ice should form in all cells (i.e., PIF = 1) if they are frozen to low enough temperature, which is not necessary tenable according to several recent experimental observations [14,26,27]. Therefore, it is of significance to further improve the PIF model for better prediction and understanding of ice nucleation in living cells during freezing.

To achieve this goal, we present a modified PIF model in this study by incorporating a new parameter, the critical cell volume (*V_f_*), into the Toner's model. To evaluate the validity and accuracy of the modified PIF model, an extensive set of experiments were conducted to study the freezing response of HeLa cells in terms of both water transport across the cell plasma membrane and IIF. The mechanisms of ice nucleation and their dependence on key parameters such as temperature, cooling rate, and membrane permeability (to water) of HeLa cells were quantitatively investigated. Moreover, we reveal that the critical volume is different for the SCN and VCN mechanisms. This new model will be a valuable tool for understanding and predicting ice nucleation in living cells during freezing for both cryopreservation and cryotherapy applications.

## Materials and Methods

### Cells and Reagents

HeLa cells were obtained from Prof. Longping Wen (USTC, China) as a gift. Dulbecco's modified eagle's medium (DMEM), fetal bovine serum (FBS), penicillin G, and streptomycin were purchased from HyClone (Thermo Fisher Scientific Inc., USA). Trypsin was purchased from Biosharp (Biosharp Co., Ltd, China).

### Cell Culture and Sample Preparation

HeLa cells were maintained in culture medium (90% DMEM) supplemented with 10% FBS, 100 U L^−1^penicillin G, and 100 U L^−1^streptomycin. The cells were cultured at 37°C in 5% CO_2_ environment. After reaching ∼90% confluence, the culture plate to which the HeLa cells adhered was washed twice with 1× phosphate-buffered saline (PBS). After 5 minutes of trypsinization (in 2 ml trypsin), HeLa cells were centrifuged (100 × g) for 5 min and re-suspended in 1× PBS for further experiments.

### Cryomicroscopy Studies of Transmembrane Water Transport and IIF

**Figure 1:**
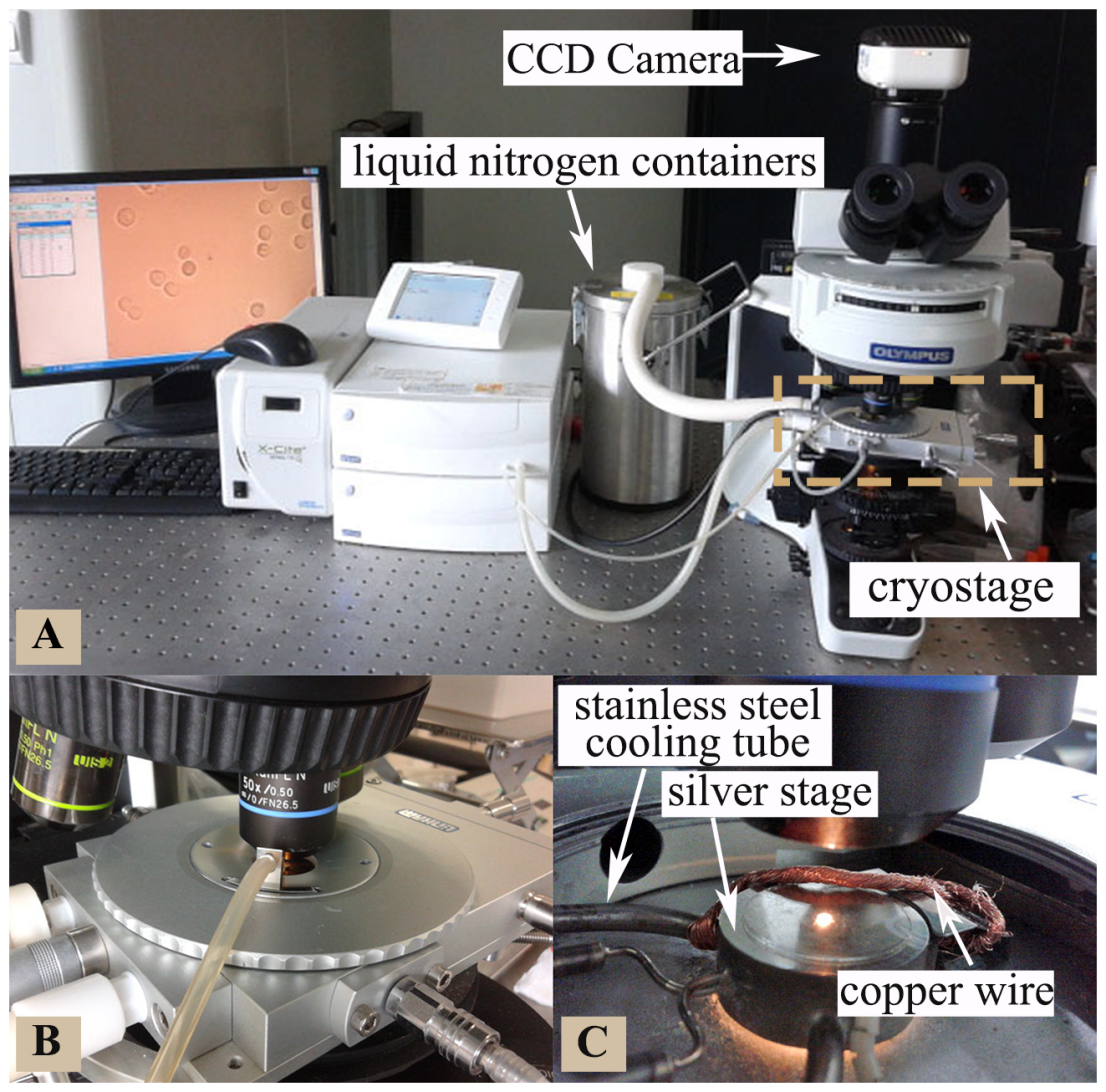
The cryomicroscopy system used for experimental studies. (A) an overview of the whole setup including the computer, Olympus BX53 microscope, CCD camera, and FDCS196 cryostage assembly, (B) a close-up view of the cryostage assembly, and (C) an inside view of the silver cryostage for cooling/warming together with the copper wire for seeding extracellular ice.

As illustrated in Fig. 1, the cryomicroscopy system consists of the Olympus BX53 optical microscope mounted with a temperature-controllable FDCS196 cryostage (−196°C to 125°C) assembly. The control accuracy of temperature, cooling/warming rates, and holding time are ±0.1°C, ±1°C min^−1^, and ±1 s, respectively (provided by the product manual). The sample chamber was filled with nitrogen vapor during the full course of experiments to minimize condensation on the glass window for visualization. A 50× long working distance objective and a Qimaging (Survey, BC, Canada) MicroPublisher 5.0 RTV CCD camera were used for real-time monitoring and recording of all experimental data.

For all cryomicroscopy experiments, a small drop (10 μL) of cell suspension was pipetted onto the center of a coverslip. Another coverslip was then gently applied on top. Since the cells were observed to flow under the coverslip, we inferred that the cells were not compressed by the coverslips presumably because the thickness of the solution film between the two coverslips was greater than the diameter of the cells. HeLa cells were cooled from room temperature to −1°C at 10°C min^−1^, followed by holding at the temperature for ∼30 s to seed extracellular ice using copper wires with one end winding around the stainless steel cooling tube (as depicted in Fig. 1c). To minimize the initial cell dehydration during the ice-seeding process, the cells were warmed up after ice-seeding to −0.5°C at 10°C min^−1^. After equilibrating at −0.5°C for 3 min, the cells were cooled to a deep subzero temperature (−50°C or −100°C) at various cooling rates. Finally, the cells were heated to 40°C. Although the freezing point of the PBS solution is around −0.5°C, 3 min of equilibrium is not enough for the extracellular ice to melt completely. The sizes of the extracellular crystals are comparable to those of the cells.

Five cooling rates (5, 10, 15, 30, and 60°C min^−1^) were used to quantitatively study the freezing-induced transmembrane water transport of HeLa cells at temperatures ranging from −0.5°C to −45°C. For each cooling rate, spherical-shaped cells (n ≥28) with clear boundary were analyzed to obtain the average volumetric information at various temperatures. Image analysis was performed using gray histogram threshold segmentation with OpenCV (Open Source Computer Vision Library). The total pixel area inside a cell was tallied using the built-in function in OpenCV and then converted to square micrometers. The obtained cell area was then used to calculate the equivalent radius of the cell. Cell volume was further estimated using the equivalent cell radius.

Four cooling rates (45, 60, 75, and 100°C min^−1^) were used to study IIF in HeLa cells at temperatures ranging from −0.5°C to −50°C. For each cooling rate, at least 165 cells with clear boundary were analyzed to obtain the IIF information for estimating PIF in the cells. Additional PIF data (60 cells) for freezing at 60°C min^−1^ from −0.5°C to −100°C were obtained to validate the effectiveness of the modified PIF models.

The experimental data of transmembrane water transport and IIF were then fitted using mathematical models to extract the model parameters for further prediction and understanding the ice formation process. The fitting was done by pooling all the experimental data of cell volume (or IIF) obtained at the different cooling rates together that are fitted simultaneously using the water transport (or IIF) model, which is therefore called “pooled fitting” in this study. Detailed information of the models is given below.

### Modeling of Water Transport during Freezing

Transmembrane water transport or cell dehydration during freezing at various cooling rates was modeled using the following equations [14,25,28,29]:
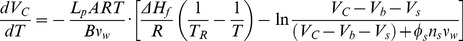
(1)

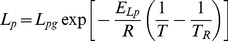
(2)where *V_C_* is cell volume, *A* is surface area of the cell, *R* is the universal gas constant, Δ*H_f_* is molar heat of fusion of water, *V_b_* is osmotic inactive volume of the cell, *φ_s_* is dissociation constant of salt, *n_s_* is molar amount of intracellular salt, *ν_w_* is partial molar volume of water, *T_R_* is reference temperature (273.15K), *L_p_* is water permeability of the cell plasma membrane, *L_pg_* is water permeability of the cell plasma membrane at *T_R_*, and *E_Lp_* is activation energy for water transport across the cell plasma membrane. This model is based on the assumption that cell membrane is permeable only to water and Δ*H_f_* is independent of temperature. The model parameters including *L_pg_*, *E_Lp_*, and *V_b_* were determined by “pooled fitting” the model to our experimental data of cell volume obtained from the cryomicroscopy studies. The *L_pg_* and *E_Lp_* were then further utilized to predict the cell volume and surface area, which are needed to predict the probability of ice formation (PIF) in cells using the PIF model detailed below.

### Modeling of PIF in Cells during Freezing

The probability of ice formation (PIF) in cells accounting for both SCN and VCN is as follows [15,25]:
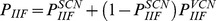
(3)

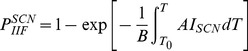
(4)

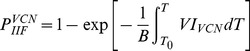
(5)where *B* is cooling rate in °C min^−1^, *T*_0_ is equilibrium freezing temperature of isotonic solutions (−0.5°C), *A* and *V* are cell surface area and volume (that can be determined from the transmembrane water transport studies), respectively, and *I^SCN^* and *I^VCN^* are nucleation rates due to SCN and VCN, respectively, which have been widely estimated using the Toner's original model as follows [15]:
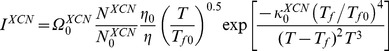
(6)where the superscript XCN represents the nucleation mechanism for SCN or VCN, *N* is number of water molecules in contact with the plasma membrane (for SCN) or in the cells (for VCN), *T_f_* is equilibrium freezing temperature of cytoplasm, the subscript ‘0’ refers to isotonic condition, *Ω_o_^XCN^* and *κ_o_^XCN^* are kinetic and thermodynamic parameters of IIF, and *η* is viscosity of the cytoplasm. In order to give a good fitting to our experimental data of IIF, we further modified the Toner's original model for nucleation rates by introducing a critical volume (*V_f_*) into Eq. 6 as follows:
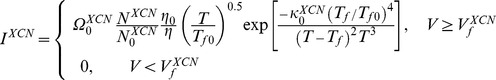
(7)


The viscosity of the cytoplasm can be estimated using the free volume model as follows [25,30]:
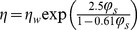
(8)

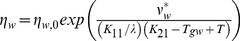
(9)where *φ_s_* is volume fraction of salts, *η_w_* is viscosity of water, *η_w,o_* is pre-exponential constant, 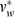 is specific volume of water at 0 K, *K*_11_/*λ* and *K*_21_ are two free volume parameters for water, and *T_gw_* is glass transition temperature of water. The equilibrium freezing temperature *T_f_* is defined as the following,
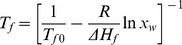
(10)where *x_w_* is water mole fraction in the cytosol.

The model parameters including *Ω_o_*, *κ_o_*, and *V_f_* for SCN and VCN were determined by “pooled fitting” the model to our experimental data of IIF obtained from the cryomicroscopy studies.

## Results

### Transmembrane Water Transport

**Figure 2:**
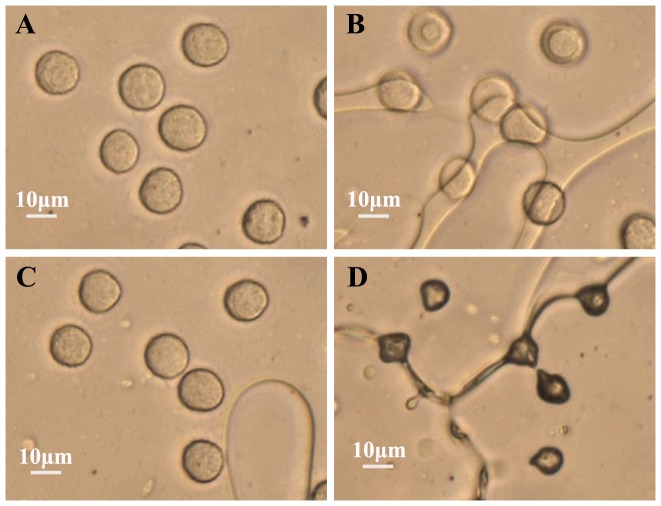
Typical images showing morphological change of HeLa cells before and after freezing. (A) at room temperature (21.6°C), (B) after seeding extracellular ice at −1°C, (D) at −0.5°C after ice-seeding and warming back, and (D) after further cooling at 30°C min^−1^ to −19.2°C.

Typical images of HeLa cells at room temperature, after ice seeding at −1°C, after seeding ice and warming back to −0.5°C, and after further cooling down to −19.2°C at 30°C min^−1^ are shown in Fig. 2a, b, c, and d, respectively. When ice was seeded using the copper wire, the differences in chemical potential of water across the cell membrane induced exosomosis of intracellular water and apparent cell dehydration was observable (Fig. 2b *versus* a). After heating back and equilibrating the cells for three minutes at −0.5°C (Fig. 2c), their size and shape restored due to melting of most of the extracellular ice [25]. When further freezing to −19.2°C, extensive dehydration of the cells is clearly visible (Fig. 2d).

**Figure 3:**
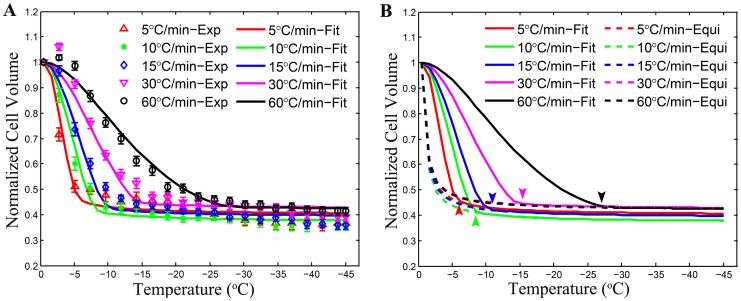
Quantitative data of normalized cell volume during freezing at cooling rates of 5, 10, 15, 30, and 60°C min^−1^. (A) average experimental data (symbols, n≥28) together with pooled model fitting results (lines, *R^2^* = 0.97), (B) a comparison between the predicted equilibrium curves (dotted lines) and the model fitting curves (solid lines) where the arrows indicate the position where the transmembrane water transport is in the equilibrium. The error bars represent standard error of the mean (SEM).

The data of normalized cell volume and the corresponding “pooled fitting” results at five different cooling rates are shown in Fig. 3a. The initial volume that we used here is the average volume at the equilibrium temperature (−0.5°C) after ice-seeding, which was done because cells move and slightly deform during seeding ice in the extracellular solution and it was difficult to identify a specific cell before and after ice-seeding. The average radius of HeLa cells at −0.5°C was measured in this study to be 7.51±1.21 μm (n = 166). The parameters in the transmembrane water transport model were determined to be 0.1166 μm min^−1^ atm^−1^ and 11.9236 kcal mol^−1^ for *L_pg_* and *E_Lp_*, respectively (*R^2^* = 0.9702).

As shown in Fig. 3a, the curves of normalized cell volume under different cooling rates are very different from each other, indicating a strong dependence of the water transport process on cooling rate. During the initial phase of the cooling process, cells undergo rapid dehydration. As the temperature continues to decrease, the kinetics of water transport slows down and asymptotically approaches a constant value (Fig. 3b). This may be attributed to the temperature dependence of the plasma membrane permeability to water [31] since *L_p_* at −40°C is only 2.4% of its value at −0.5°C. Figure 3b also depicts the starting temperature (pointed by arrows) where the water transport process has entered the equilibrium phase, showing its strong dependence on cooling rate, as well.

### Intracellular Ice Formation

**Figure 4:**
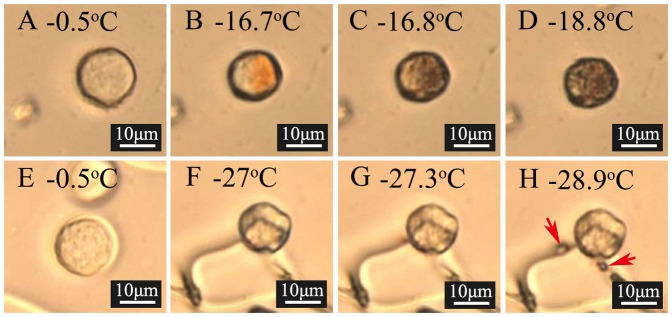
Morphological observation of intracellular ice formation (IIF) during freezing HeLa cells at 60°C min^−1^. Both “darkening” (A-D) and “twitching” (E-H) showing apparent IIF were observed. Red arrows in (H) indicate the gas bubbles seeping out of the cell.

Two types of apparent intracellular ice formation during freezing were observed in the present study with HeLa cells (Fig. 4). The darkening IIF occurred with a clear change in gray level in the cells, resulting in sudden darkening of the cells (Fig. 4b-d), indicating the instantaneous propagation of the intracellular ice once ice embryos were nucleated. In contrast, the twitching IIF only exhibited a sudden trembling movement that was observable under microscope with minimal change in gray level (Fig. 4e-h). As illustrated in Fig. 4h, bubble-like intracellular materials (red arrows) were observable to seep out of cells after the twitching movement, possibly due to IIF induced damage to the cell plasma membrane and the subsequent leakage of cytoplasm out of the cells. This phenomenon was also observed in cells undergoing darkening IIF.

**Figure 5:**
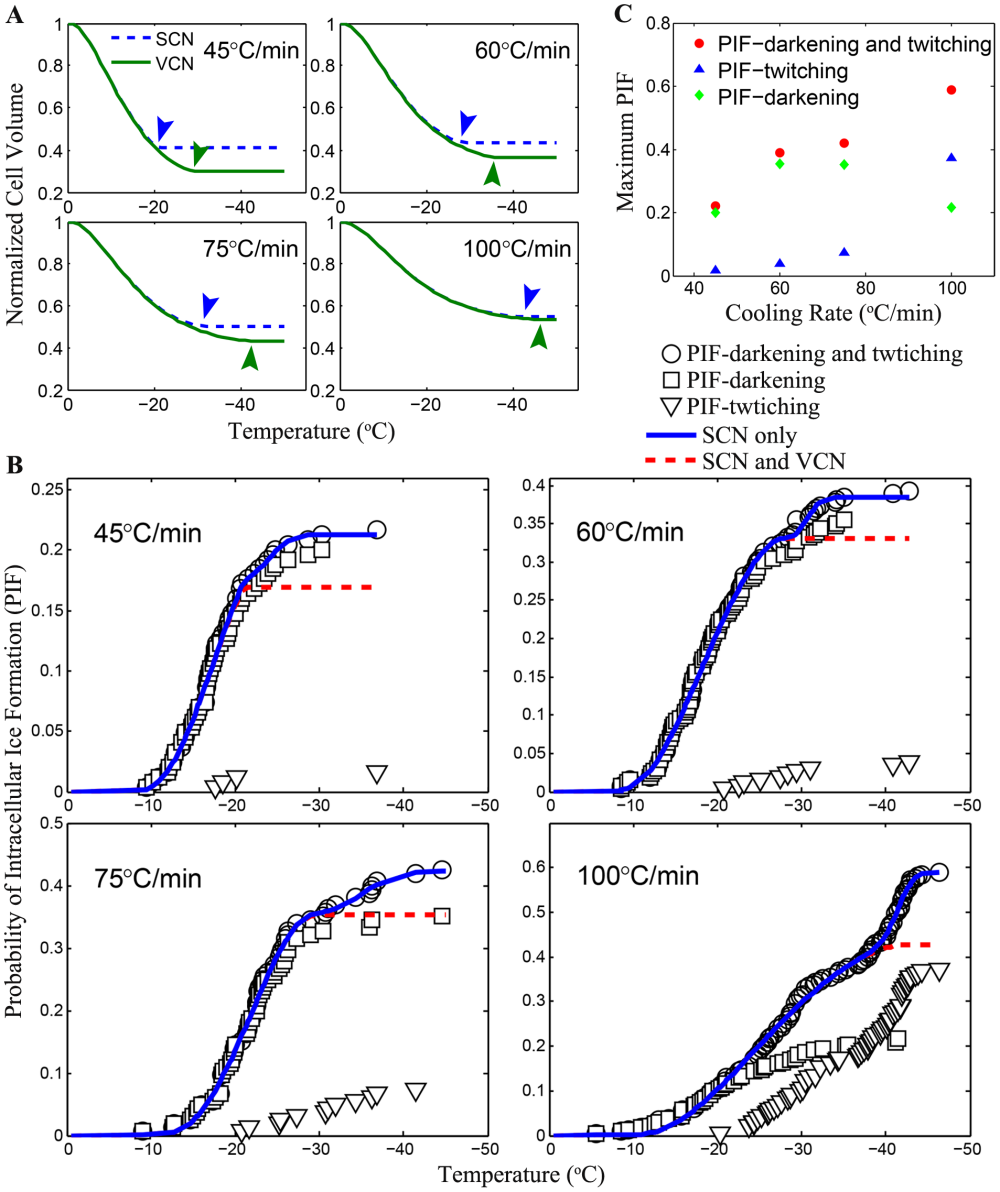
Quantification of PIF in HeLa cells during freezing. (A) calculated normalized cell volume of HeLa cells during freezing using model parameters determined from the water transport studies where arrows indicate the starting points of the critical volumes *V_f_^XCN^*, (B) probability of ice formation in HeLa cells appeared as darkening (square), twitching (triangle), and both (circle) at cooling rates together with model fit assuming SCN only (dotted line) and both SCN and VCN (solid line), and (C) the dependence on cooling rate of maximum PIF appeared as darkening (square), twitching (triangle), and both (circle) in HeLa cells.

To verify the validity of the modified PIF model, the parameters for transmembrane water transport across the cell membrane were utilized to predict the cell volume under freezing at four different cooling rates (Fig. 5a), which was used for simultaneous fitting to the experimental PIF data with the modified PIF model and the results are shown in Fig. 5b. The critical volume together with the kinetic and thermodynamic parameters for both SCN and VCN were determined and are given in Table 1. As shown in Fig. 5b, it appears that darkening dominated twitching at slower cooling rates while at higher cooling rate (100°C min^−1^), twitching dominated.

**Table 1:** Ice nucleation parameters estimated using both original and modified PIF model: O refers to the original PIF model, M refers to the modified version, and N/A represents not applicable.

*B*	Model	*Ω_o_^SCN^*	*κ_o_^SCN^*	*Ω_o_^VCN^*	*κ_o_^VCN^*	*V_f_^SCN^*	*V_f_^VCN^*
(°C min^−1^)		(m^−2^ s^−1^)	(K^5^)	(m^−2^ s^−1^)	(K^5^)		
45	O	3.22×10^8^	5.53×10^9^	1.08×10^9^	6.26×10^10^	N/A	N/A
	M	2.70×10^8^	5.20×10^9^	5.99×10^8^	5.32×10^10^	0.41*V_0_*	0.30*V_0_*
60	O	2.54×10^8^	4.25×10^9^	1.58×10^20^	5.24×10^11^	N/A	N/A
	M	2.55×10^8^	4.26×10^9^	1.00×10^20^	5.15×10^11^	0.43*V_0_*	0.36*V_0_*
75	O	8.22×10^8^	9.61×10^9^	8.10×10^19^	6.49×10^11^	N/A	N/A
	M	8.44×10^8^	9.70×10^9^	6.38×10^11^	2.45×10^11^	0.50*V_0_*	0.43*V_0_*
100	O	2.62×10^8^	6.33×10^9^	2.30×10^27^	1.24×10^12^	N/A	N/A
	M	2.61×10^8^	6.32×10^9^	8.09×10^26^	1.21×10^12^	0.55*V_0_*	0.54*V_0_*

According to the experimentally obtained maximum PIFs at cooling rates of 45, 60, 75 and 100°C min^−1^ (Fig. 5c), the overall PIF and twitching PIF appears to increase along with the cooling rate. In contrast, the darkening PIF exhibits an inverted parabolic shape, where the probability reaches its maximum value of ∼40% between 60 and 75°C min^−1^ for HeLa cells.

**Figure 6:**
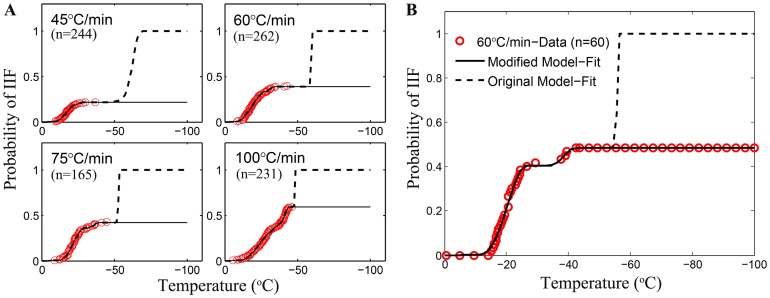
A comparison of the modified PIF model and the original one for predicting IIF in HeLa cells during freezing at four different cooling rates. (A) Solid lines represent the prection of modified PIF model whereas the dotted lines represent that of the original model. The red circles denote experimental data of the PIF accounting for both darkening and twitching. (B) Extra experiments to further validate the efficiency of the two models. Symbols indicate the experimental data. “n” denotes the cell numbers.

To show the importance of introducing the critical volume in the modified PIF model, the experimental PIF data obtained under different cooling rates (45, 60, 75 and 100°C min^−1^) were fitted using both the original Toner's and the modified PIF models. Fig. 6a illustrates a comparison of the two model predictions at temperature from 0°C to −100°C, where the PIF data was observed to keep constant at the lower temperatures (−50°C to −100°C). While both models were capable of delivering accurate and logical predictions at the beginning of the cooling process with high fitting goodness (*R^2^*≥0.9963), the fitting curves plotted by the original PIF model rapidly increase to 100% in the deep subzero region. In contrast, the modified PIF model gives more accurate prediction of PIF for HeLa cells over the entire temperature range. The data from additional experiments for freezing at 60°C/min from −0.5°C to −100°C shown in Fig. 6b confirm that no IIF would occur below −50°C and validate the effectiveness of the modified PIF model for accurate prediction of IIF over a wide range of temperature. Note that a few of ice nucleus may still form while not grow to a detectable dimension because of the extremely lower diffusion coefficient. The concentrated residual intracellular solution will be vitrified at the lower temperatures. Since the PIF model we used is phenomenological, we omit such undetectable ice nuleation in our statistics.

The ice nucleation parameters obtained from the curve fitting to data in Fig. 6a using the original and modified PIF model are shown in Table 1. All SCN related parameters generally appears to be in good agreement for the two models. However, a large difference is noticeable in terms of the model parameters for VCN.

## Discussion

### Transmembrane Water Transport

Since cell volume and area are required for predicting PIF (Eqs. 4-7), it is important to study cell dehydration as a result of transmembrane water transport during freezing for investigating IIF. Therefore, the dehydration responses of HeLa cells at five different cooling rates were experimentally studied and the experimental data were fitted using the transmembrane water transport model (Eqs. 1–2) to determine *L_pg_*, *E_Lp_*, and *V_b_* for further predicting the cell volume and area during freezing at any cooling rates. Although the two parameters (0.1166 μm min^−1^ atm^−1^ for *L_pg_* and 11.9236 kcal mol^−1^ for *E_Lp_*) obtained for HeLa cells are not the same as that reported in a previous study [32], the differences (37.23% for *L_pg_* and 8.97% for *E_Lp_*) are not huge either. In addition, our data are considered to be more accurate and suitable for the subsequent prediction of PIF because they were obtained by “pooled fitting” to experimental data obtained with five different cooling rates while in the previous study, the measurements were done under only one cooling rate.

As shown in Fig. 3a, there are one and two data points at temperatures between −3 and −7°C for the cooling rates of 30 and 60°C min^−1^, respectively, for which the model could not give a good fit. This unusual increase in cell volume during the initial stage of freezing was probably an artifact due to the unclear boundary of cells for determining cell volume using imaging processing with OpenCV, which was a result of freezing induced concentration of extracellular solution that were pushed together around the cells by extracellular ice formed at these two high cooling rates (data not shown). However, no significant effect on the fitting results of model parameters (*L_pg_*, *E_Lp_*, and *V_b_*) were observed, probably because the impact of these few data points were minimized by the many other data points as a result of the use of the “pooled fitting” methods to extract the two model parameters for transmembrane water transport.

### Intracellular Ice Formation

Both the twitching and darkening phenomena associated with IIF were observed in the study. As previously reported, the twitching phenomenon was observed to occur as a sudden tremble with minimal change in gray level in cells whereas the darkening event was found to exhibit as either ice formation propagated from one side of the plasma membrane to the other or a sudden opacity flashing throughout the cell [15,16]. The number of twitching occurrence was found to increase along with the cooling rate (Fig. 5c). Faster cooling rate may cause the presence of a higher supercooled degree of intracellular solution, which might result in the formation of fine ice crystals that are transparent to light and therefore, not easily observable under microscope [16]. Consequently, only a brief tremble of the cell is noticeable. The darkening phenomenon of IIF, on the other hand, exhibits an inverted parabolic trend where the probability reaches a maximum value of ∼40% between 60 and 75°C min^−1^ for HeLa cells. This phenomenon suggests that the darkening IIF may be associated with large intracellular ice crystals that can block light from going through them, resulting in a dark appearance in the area occupied by them. Twitching IIF is cell type dependent because the twitching IIF in HeLa cells was found to occur from ∼−20°C to −45°C which covers the homogeneous nucleation temperature (∼−40°C) while the twitching IIF of granulocytes [16] was found to occur 6.3–7.4°C above the homogeneous nucleation temperature. Therefore, our finding does not support the hypothesis that twitching IIF is induced only by the VCN mechanism [15,16]. Nevertheless, the observations that darkening IIF initiates from one side of the plasma membrane (Fig. 4) and the incidence of darkening cells is mainly between −10°C and −35°C (Fig. 5c) suggest darkening IIF may be mainly associated with SCN.

The maximum PIF due to both SCN and VCN in HeLa cells were found to be less than 0.5 during freezing at cooling rates below 75°C min^−1^. This may contribute to the insufficient destruction of cancer cells in the tumor peripheral where the cooling rate is usually less than 50°C min^−1^ during cryoablation using a single cryoprobe [33]. Moreover, as shown in Fig. 6, the original model reported by Toner et al. [15] for PIF was unable to predict PIF in cells if the maximum PIF is less than 1. We overcome this problem by modifying the original model using a critical volume (*V_f_*) below which the nucleation rate due to either SCN or VCN is zero (Eq. 7). Interestingly, the *V_f_^SCN^* is constantly higher than *V_f_^VCN^* (Fig. 5a and Table 1) and a change in slope is clearly noticeable in the experimental data of PIF (summation of both twitching and darkening) where the predicted PIF due to SCN only reaches maximum. It could be inferred from these observations that the VCN mechanism might not be a major contributor to PIF only after the SCN mechanism stops. In other words, intracellular ice during freezing is first nucleated primarily by SCN induced by extracellular ice through the cell plasma membrane when the cell volume is above *V_f_^SCN^*. When the cell dehydrates with a volume less than *V_f_^SCN^*, nucleation of intracellular water due to SCN diminishes and may be catalyzed in the whole cellular space heterogeneously by the internal organelles and/or homogeneously by the stochastic thermodynamics when the cell volume is above *V_f_^VCN^*. This assumption is in good agreement with the observation [15] that VCN is mostly found in the absence of external ice. Here, our interpretation may explain why both SCN and VCN mechanisms could occur in the presence of extracellular ice albeit they may occur sequentially rather than in parallel.

## Summary and Conclusions

In this study, the transmembrane water transport and IIF in HeLa cells were thoroughly investigated both experimentally and by modeling. Our modified PIF model is shown to be efficient in predicting PIF over a wide range of temperature regardless of the value of the maximum PIF while the original PIF model might give a misleading prediction of PIF if the maximum PIF is less than 1. The critical volume that we introduced in the modified model is found to increase with the increase of cooling rate from 45 to 100°C min^−1^. From the data of the critical volume and the change in slope of the PIF data, we infer that the IIF due to VCN mainly occurs after IIF as a result of SCN stops. The new PIF model for nucleation-limited IIF together with the concept of critical volume introduced in this study may be valuable for understanding the mechanism and kinetics of ice formation in living cells and optimizing the protocols of both cryopreservation and cryotherapy applications.

## Supporting Information

Video S1 **Video for twitching IIF.**
(AVI)

File S1 **Raw data of the normalized cell volume.**
(ZIP)

File S2 **Raw data of probability of IIF (PIF).**
(ZIP)
